# Mind the Localized Skeletal Pain: Chronic Recurrent Multifocal Osteomyelitis

**DOI:** 10.7759/cureus.15101

**Published:** 2021-05-18

**Authors:** Aung Than, Haresh Tulsidas

**Affiliations:** 1 Internal Medicine, Singapore General Hospital, Singapore, SGP

**Keywords:** chronic recurrent multifocal osteomyelitis, idiopathic, aseptic, inflammatory bone disorder, immunosuppressant therapy

## Abstract

Chronic recurrent multifocal osteomyelitis (CRMO) is a rare idiopathic aseptic inflammatory bone disorder affecting primarily children and adolescents characterized by an insidious onset of pain, swelling, and tenderness over the affected bones. The clinical signs and symptoms of CRMO are nonspecific, radiological and histopathological tests are essential for its diagnosis.

We present a case of an 18-year-old young man who was diagnosed with CRMO by a combination of clinical data, laboratory results, radiological imaging, and bone biopsy. The patient started anti-inflammatory and immunosuppressant therapy, and his lower extremity pain and swelling improved.

This report highlights to investigate promptly in children and adolescents with chronic leg pain, to emphasize the importance of combined clinical, laboratory, and imaging tests for early identification, to have a greater understanding of the imaging appearance and increasing knowledge of this condition, which help shorten time to reach a diagnosis and prevent permanent osseous damage and long-term disabilities.

## Introduction

Chronic recurrent multifocal osteomyelitis (CRMO) also known as chronic nonbacterial osteomyelitis (CNO) is a non-infectious autoinflammatory bone disease affecting primarily children and adolescents. It is characterized by an insidious onset of pain, swelling, and tenderness localized over the affected bones. We report the case who presented with chronic bilateral leg swelling and pain subsequently found to have CRMO after a combination of clinical, laboratory, and radiological data and biopsy.

## Case presentation

An 18-year-old young man was referred to our internal medicine clinic for bilateral leg swelling. During the routine medical check-up, he was found to have lower extremity swelling and directed to a vascular surgeon for possible chronic venous insufficiency. The vascular clinic confirmed his venous system was competent, excluded venous reflux and venous obstruction after bilateral venous duplex ultrasonography.

On visiting our clinic, he reported having bilateral lower limb swelling for ten years. He had minimal swelling of his legs in the morning. By the end of the day, he noted swelling of legs up to the distal third of both legs associated with pain. He experienced lower leg pain that began with running activity. The pain scale was quantified as 4 out of 10. It increased with continued exertion, resolved with rest, or when he stopped engaging in activities. He was asymptomatic at rest and during the activity of daily living.

He denied early morning stiffness, back pain, or positive family history. He tried massage therapy, footwear modifications, and over-the-counter analgesics as needed. Recently he observed more swollen ankles associated with pain on activity. His bowel habit is regular with no diarrhea, abdominal pain or weight loss, or skin rashes. He did not have a cough, fever, or night sweating. There was no significant family history of autoimmune disease and bone disease. 

On examination, he was able to walk with a normal gait. There was bilateral ankle nonpitting edema with no tenderness, no increase of the local temperature, and peripheral pulses were present. Fine tremors of the hands and sweaty palms were noted. The rest of the examination was unremarkable. At that time, clinical impressions were likely hyperthyroidism and bilateral ankle arthropathy of uncertain etiology.

**Figure 1:**
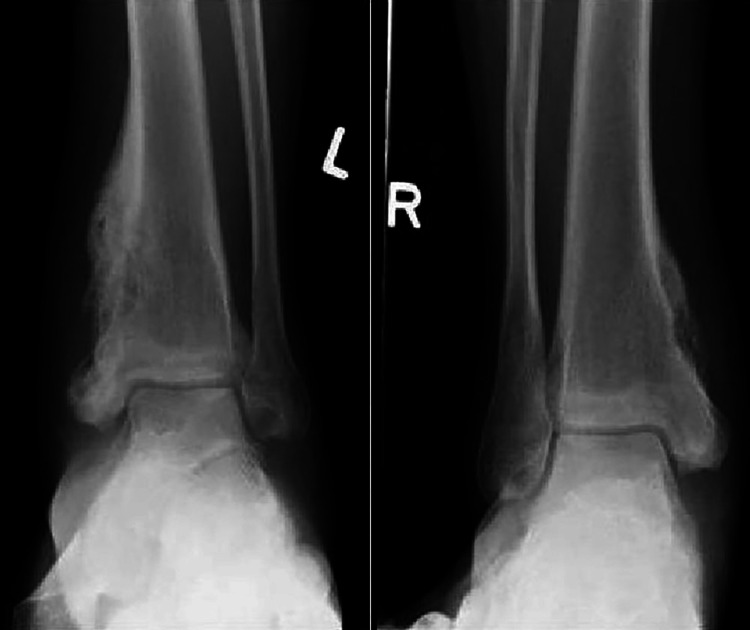
Left and right X-ray of the ankle. There is a periosteal reaction seen along with the medial aspects of bilateral distal tibial meta-diaphysis. This is more florid on the left. There is also sclerosis of the left calcaneus with subtle periosteal reaction at the plantar cortex.

Laboratory tests disclosed he was biochemically hyperthyroid, free thyroxine 16 pmol/L ( 8.8-14.4 pmol/L ) and Thyroid Stimulating Hormone (TSH) 0.588 MU/L ( 0.65-3.70 MU/L ). Complete blood count, renal panel, liver panel, serum calcium, urine protein creatinine ratio all were in the normal range and Chest X-ray finding was also normal. Radiographic findings of ankles (Figure 1) revealed periosteal reaction seen along with the medial aspects of bilateral distal tibial meta-diaphysis. Soft tissue swelling was noted around the ankles bilaterally.

**Figure 2:**
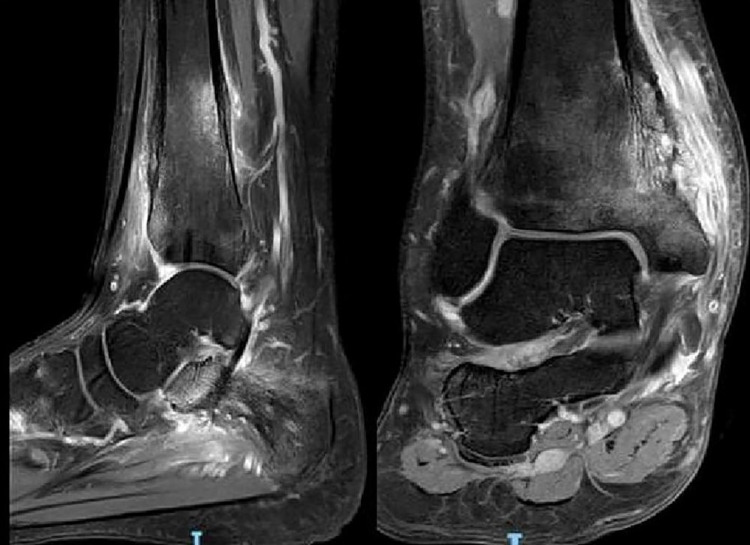
MRI of Left and right ankle. Erosions along the medial cortex of the distal tibial metaphysis and epiphysis with florid periosteal reaction and marked surrounding soft tissue edema. Bony edema also noted within the talus, calcaneum, navicular, medial cuneiform and base of first metatarsal. Findings are fairly symmetrical.

On reviewing him a month later, he reported well. He was not given antibiotics. He had occasional pain in the ankles on long-standing and prolonged walking. The repeated TSH and free thyroxine tests turned out normal. Triiodothyronine and thyroid-stimulating receptor antibody levels were normal. An abnormal X-ray finding of the ankle prompted him to go for further investigations. Inflammatory marker levels were non-significant (erythrocyte sedimentation rate [ESR] 10 mm/hour [1-10 mm/hour], C-reactive protein [CRP] 10.3 mg/L [0.2-9.1 mg/L]). Antinuclear antibody, extractable nuclear antigen (ENA) antibodies screening, and antineutrophil cytoplasmic antibody tests were negative. MRI of the ankles (Figure 2) revealed erosions along the medial cortex of the distal tibial metaphysis and epiphysis with periosteal reaction and marked surrounding soft tissue edema. Bony edema was also noted within the talus, calcaneus, navicular, medial cuneiform, and base of the first metatarsal. Findings were fairly symmetrical.

**Figure 3:**
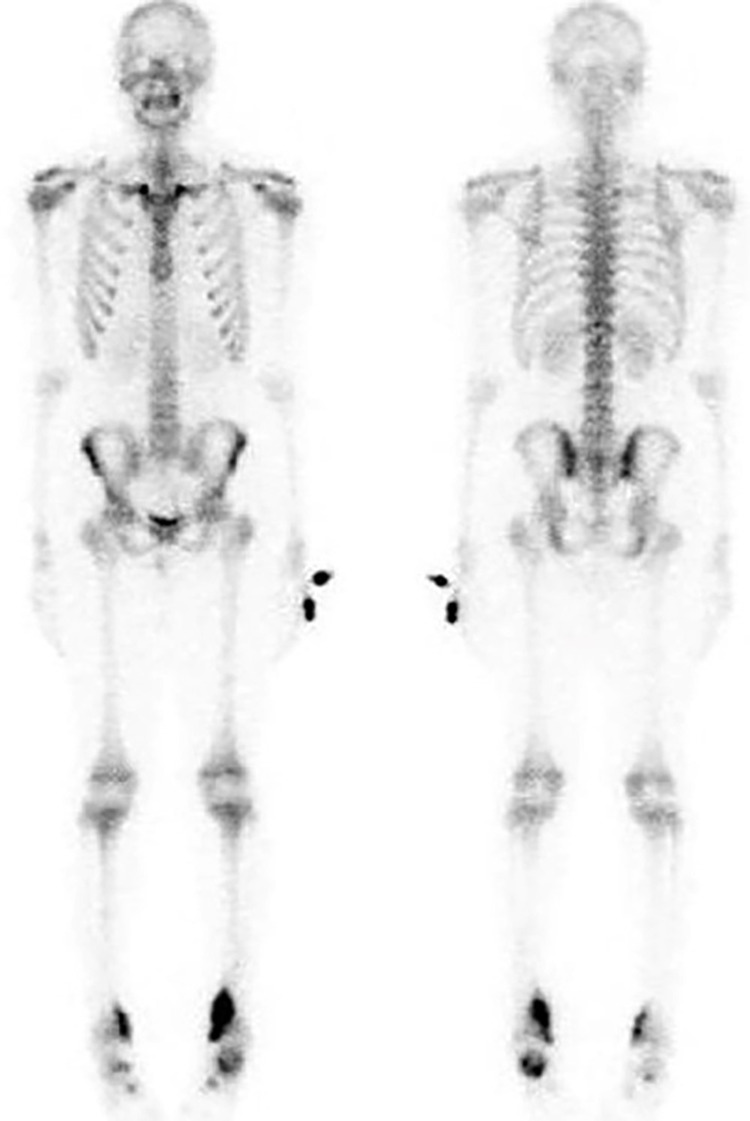
Bone scintigraphy. Increased uptake in bilateral distal tibia, calcaneum, and midfoot bones corresponding to prior MRI findings, are in keeping with infective/inflammatory changes. No suspicious focus of increased radiotracer uptake is seen in the rest of the skeletal system to suggest disease involvement.

On following clinic visit, he experienced ankle pain at night which was worse on the left side. Given the relatively prolonged clinical course over several years, bland biochemistry tests, combined with the multifocal osteolytic bone lesions on imaging, the provisional diagnosis of CRMO was made. He was given indomethacin for pain control. A radioisotope bone scintigraphy (Figure 3) was arranged to detect other silent lesions. Increased uptake in bilateral distal tibia, calcaneum, and midfoot bones corresponding to prior MRI findings was observed on bone scan in keeping with inflammatory bone disease. No suspicious focus of increased radiotracer uptake is seen in the rest of the skeletal system.

Screening test for tuberculosis (TB) is important to detect latent disease before consideration of starting biologic or disease-modifying anti-rheumatic drugs. TB QuantiFERON test was positive. Sputum for acid-fast bacilli (AFB) smear, culture, and TB nucleic acid amplification test all were negative. Subsequently, a bone biopsy was performed to exclude chronic infections (like bacterial osteomyelitis, TB) and malignancy (bone tumors, leukemia, lymphoma, Langerhans cell histiocytosis) of bone. The left medial malleolus core biopsies showed compact bone, minimal intertrabecular areas with the presence of inflammatory cells. There were no granulomas, no malignant cells identified. No AFB were seen on the Ziehl-Neelsen stain and bone culture was sterile.

CRMO can be a difficult diagnosis as there are no validated diagnostic criteria for CRMO. Based on the clinical, radiological, histopathological findings and sterile bone culture, diagnosis of CRMO was subsequently established. In view of the findings on MRI ankles and bone formation on biopsy, methotrexate was initiated to reduce symptoms as well as prevent further osseous expansion. He was also treated for latent TB. He was feeling well with no more ankle pain. He continues to have close follow-up arrangements.

## Discussion

CRMO is a rare disease, likely to be underdiagnosed and underreported due to unawareness in medical practice. There are insufficient data to determine the true prevalence of the CRMO, and over 500 existing patients are reported worldwide based on case series studies [1]. It mostly affects children and adolescents and the peak onset is the age between 7 and 12 years. The pathogenetic mechanism of the sterile bone inflammation remains unknown. Studies suggest that bone inflammation may be the result of an imbalance between pro- and anti-inflammatory cytokine expression from innate immune cells and subsequent osteoclast differentiation and activation, resulting in osteolytic lesions [2]. Environmental and genetic factors, as reported by finding of CRMO in siblings and positive family history of autoimmune diseases, may interplay with the underlying bone inflammation [3]. Infections are not involved in disease etiology. No infectious agents are detectable at the site of the bone lesion.

CRMO is characterized by the insidious onset of pain and swelling localized over the affected bones. Some of the patients complained of nocturnal bone pain which may be misinterpreted as growing pain or non-malignant bone tumor. The symptoms of CRMO can be variable from asymptomatic inflammation of single bones to chronic, recurrent multifocal osteitis. The bone lesions are typically located in the metaphyses of long bones of lower extremities near knees and ankles. Symmetric involvement is common. The other frequent locations include pelvis, spine, clavicle, and long bone of upper extremity. Patients generally appear well but may experience concomitant systemic symptoms including low-grade fever and generalized malaise.

Physical examinations are mostly unremarkable. There may be localized tenderness and warmth over the affected bones. CRMO may be accompanied by other extra-osseous inflammatory disorders such as pustulosis palmoplantaris, acne, psoriasis, inflammatory bowel disease (IBD), spondyloarthropathy, and infrequently pyoderma gangrenosum, Sweet syndrome, or granulomatosis with polyangiitis. 

CRMO remains a diagnosis of exclusion. It is essential to rule out other more common causes of recurrent inflammation such as infection, malignancy, and autoimmune disorders. In most CRMO patients complete blood count is usually normal, and inflammatory markers such as CRP, ESR may be mildly raised. All cultures of blood and tissue specimens are negative. Many patients were initially treated for bacterial osteomyelitis due to similarity in presentation with bacterial acute osteomyelitis. Imaging techniques are crucial for the diagnosis of CRMO. Plain X-ray usually discloses cortical irregularities, osteolytic destruction with surrounding sclerosis in the metaphysis areas of long bones.

Our case had the typical location of chronic symmetric ankle swelling and pain, but no systemic symptoms and no associated extra-osseous disorders. Initial findings of lytic lesion on plain X-ray of ankles directed him to proceed with further workup including MRI, bone scan, and bone biopsy.

MRI can demonstrate marrow edema associated with periostitis, soft-tissue inflammation. It can also locate asymptomatic silent lesions. Whole-body MRI, a gold standard diagnostic modality, allows detection of disease extent of CRMO, facilitates monitoring of treatment efficacy [4]. Although the sensitivity of bone scintigraphy is lower than MRI, bone scintigraphy may be used alternatively to identify all silent lesions in the whole body. There is no specific feature in bone biopsy for CRMO. Histologic findings are consistent with chronic inflammation which include destruction of normal bone structure and presence of nonspecific inflammatory cells. Bone biopsies are usually performed to exclude chronic infections such as bacterial osteomyelitis, TB, and malignancies in particular leukemia, lymphoma, bone tumors.

A combination of clinically well-looking patient, chronic bone pain, normal laboratory results, symmetrical bony lesions on imaging, culture-negative biopsies shortened the length of time to the diagnosis of our patient. Our patient had more than the threshold diagnostic criteria for CRMO by Jansson’s by the presence of two major features such as radiologically proven osteolytic lesion, multifocal lesion and three minor requirements including normal blood count and good general health state, unremarkable CRP and ESR, and chronic duration more than six months [5]. He was doing well with non-steroidal anti-inflammatory drugs (NSAID) and methotrexate.

The goal of therapy for CRMO is to control disease activity in order to decrease disease complications and prevent skeletal damage. There is no consensus treatment of CRMO, and most evidence comes from small case series or retrospective cohorts. NSAID is the first-line treatment for all CRMO patients for symptomatic pain relief. In a prospective study, almost half of the cases achieved remission in pain and swelling within the first year of treatment with NSAID [6]. However, relapse is common, and new lesions may develop. The efficacy of NSAID therapy is associated with the initial number of affected bony lesions. The patients with a high number of bony lesions at the time of disease onset had a poor response to NSAIDs [7]. The total duration of NSAID is not clear for patients who responded to NSAID therapy.

In the Childhood Arthritis and Rheumatology Research Alliance (CARRA) meeting, the members decided that the NSAIDs should be tried for at least four weeks. The patients who still have persistent pain with local tenderness or warmth are considered non-responsive to NSAID therapy. Those non-responders require additional second-line treatment which included (1) methotrexate or sulfasalazine, (2) tumor necrosis factor (TNF)-alpha inhibitors, and (3) bisphosphonates [8].

A multidisciplinary approach involving pediatricians, physical and occupational therapists, rheumatologists, and geneticists is the most efficacious way of managing these patients. 

Early diagnosis and effective treatment may prevent or decrease long-term sequelae of the disease. Permanent skeletal damage may include vertebral compression fractures, leg-length discrepancy, and disabling bone deformity like angulation secondary to growth plate damage [9]. Earlier literature suggested that CRMO was a self-limiting disease that eventually resolved after 12-18 months in most cases. However, recent data indicate that long-term outcomes in patients with CRMO may be less favorable, 50 percent of CRMO patients develop disease flares after a median of 29 months [10]. Hence, in patients with CRMO, close follow-up is critical to monitor disease activity, treatment response and capture recurrence of the disease by a combination of clinical, laboratory markers, and radiological imaging.

## Conclusions

A rare disorder, CRMO, should be suspected as one of the differential diagnoses in children and adolescents with chronic bone pain. They should be investigated promptly. A combination of imaging techniques such as plain radiograph, MRI, and bone scintigraphy is useful in the diagnosis of CRMO. Being unfamiliar with CRMO in clinical practice, many physicians commonly misdiagnosed it as bacterial osteomyelitis, delayed in referral and diagnosis. Increasing awareness of this disease and its features may prevent unnecessary diagnostic procedures, and mistreatment with prolonged antibiotic regimens. Timely effective treatment is crucial to prevent permanent skeletal damage.
